# Development and psychometric evaluation of a self-management behaviours scale in rheumatoid arthritis patients (RA-SMBS)

**DOI:** 10.1186/s12912-023-01173-4

**Published:** 2023-02-14

**Authors:** Jinglin Chen, Yuqing Song, Lihong Ou, Xia Wang, Ying Wang, Yanling Chen, Hong Chen

**Affiliations:** 1 West China School of Nursing / West China Hospital, Sichuan University, No. 37, Guo Xue Xiang, Wu Hou District, Sichuan 610041 Chengdu, China; 2 Edson College of Nursing and Health Innovation, Arizona State University, Phoenix, AZ 85004 USA; 3 Department of Rheumatology and Immunology, West China School of Nursing, West China Hospital, Sichuan University, Chengdu, China

**Keywords:** Chinses, Psychometric evaluation, Rheumatoid arthritis, Self-management behaviours scale, Scale development

## Abstract

**Background:**

Self-management behaviours can be crucial to improving disease symptoms and health outcomes in rheumatoid arthritis (RA) patients. Currently, the tools available for measuring self-management behaviours in RA patients are either generalized for patients with chronic diseases, which lack specificity or have poor reliability in the only specific scale—self-care behaviours scale (SCBS). The aim of this study was to develop a self-management behaviours scale for RA patients and evaluate its psychometric properties.

**Methods:**

The study included two steps: scale development and the psychometric evaluation. The items were developed from a literature review, in-depth individual interviews, nominal group technique, Delphi expert consultation, and a pilot test. For the psychometric evaluation, a sample of 561 patients with RA was recruited. Item analysis, content validity, exploratory and confirmatory factor analysis, convergent and discriminant validity, and internal consistency reliability were conducted to examine the psychometric properties of the RA-SMBS.

**Results:**

The final scale consists of 23 items with 4 dimensions, including medication management, exercise and joint protection, resource utilization and emotional management, and symptom management. The content validity index was 0.78. Exploratory factor analysis explained 61.89% of the total item variance. Confirmatory factor analysis indicated that the RA-SMBS fit well. Good internal consistency reliability was demonstrated (Cronbach's alpha = 0.908), and the test–retest reliability was found to be acceptable (ICC = 0.628, r = 0.780).

**Conclusions:**

The scale has good content validity, construct validity, and internal consistency reliability. It can be used to assess the level of self-management behaviours in RA patients.

**Supplementary Information:**

The online version contains supplementary material available at 10.1186/s12912-023-01173-4.

## Background

Rheumatoid arthritis (RA) is a chronic autoimmune disease characterized by synovial inflammation. The global prevalence of RA ranges from 0.5% to 1% [1]. The prevalence of RA in China is about 0.42% and affects 5 million people [2]. RA often manifested as joint pain, stiffness, swelling, limitation of joint range of motion, and general fatigue [3]. These symptoms may lead to limited daily activities and reduced work capacity, which negatively affects the quality of life in patients with RA [4] and significantly increases healthcare costs [5]. RA is incurable and the goal of treatment is to achieve treat-to-target, control disease, reduce disability, and optimize health outcomes and quality of life [6].

Given the chronic and progressive nature of RA, several studies have demonstrated that a high level of self-management in RA patients can reduce patients’ symptoms (e.g., pain, stiffness, swelling, etc.), effectively improve the patients’ physical function and quality of life [7, 8]. Self-management support provided by healthcare providers is an effective method to prompt patients’ self-management behaviours and health outcomes [9]. Healthcare providers should learn patients’ level of self-management behaviours before providing self-management support. However, a reliable and valid scale to evaluate RA patients’ self-management behaviours is not currently available.

Lorig’s definition of self-management [10] is that ‘Whether an individual is engaging in a health-promoting activity or is living with a chronic disease, he or she is responsible for day-to-day management’. Self-management shows potential as a useful tool for preventative health care [11]. Although some RA patients are aware of the importance of medication adherence and joint exercises and following doctors’ advice on management, these self-management behaviours are not the only ones that should be taken into consideration [12]. Other aspects of self-management behaviours are easily overlooked by patients, such as, underutilisation of healthcare resources, insufficient support from family and friends, and depression due to factors (e.g., long duration of illness, alternating symptoms of remission and onset of illness) [13]. Effective self-management behaviours for RA patients play a crucial role in improving RA patients’ symptoms and health outcomes across the disease course [14]. Self-management behaviours increase patients’ confidence, knowledge, and skills about their conditions, thereby assisting in managing their health problems [15]. Engaging in self-management supports patients in taking responsibility for improving their health by engaging in positive health behaviours such as physical activity, fatigue management, medication adherence, and seeking support or advice from family and friends [16].

Validated and reliable tools are needed to evaluate the level of self-management behaviours in RA patients. However, there is limited scales to evaluate self-management behaviours of RA patients. Chronic Disease Self-Management Study Measures (CDSMS) developed by Lorig et al. is the most commonly used scale for patients with chronic diseases in the world [17, 18]. The CDSMS includes the self-efficacy scale and the self-management behaviours scale. The self-management behaviours scale includes three dimensions: stretching exercises, cognitive symptom management, and communication with the physician [17, 18]. The Patient Activation Measure (PAM) assesses knowledge, skill, and confidence of self-management among patients with chronic conditions [19]. The short self-management ability scale (SMAS-S) [20] is more applicable for older adults, especially among frail elderly patients (e.g., the elderly shortly after discharge). The scale assesses patient initiatives, investment behaviours, self-efficacy, a positive frame of mind, and other aspects of self-management ability [20]. Although these scales have good psychometric properties, the contents of these instruments are not specific for RA patients. Nadrian et al. developed Self-Care Behaviours Scale (SCBS) among patients with RA in 2019 [21]. This is the only specific scale used for RA patients, but Cronbach's alpha coefficients for the SCBS subscale is lower than 0.3, indicating poor internal consistency [21]. The scale also lacks a theoretical basis for self-management [21]. Scale development through a rigorous approach should help to improve its psychometric properties and its suitability for use in target groups [22]. The approach involves a literature review, expert consultation, careful consideration of the experience and perspectives of target groups, and a solid conceptual framework. The development of the existing scale did not incorporate the experience and perspectives of target groups and a solid conceptual framework. Although these scales can be used to evaluate RA patients’ self-management, they do not accurately reflect the muti-dimensions of RA patients’ self-management.

Given the lack of a specific scale to assess the multidimensional aspect of self-management among patients with RA, the study aimed to develop a scale to measure self-management behaviours among patients with RA and to evaluate the validity and reliability of this scale. The RA-SMBS we proposed was developed based on a solid conceptual framework, a literature review, in-depth reviews, the nominal group technique (NGT), and Delphi expert consultation.

### Conceptual framework

This scale was developed based on Lorig and Holman’s conceptual framework for self-management. Lorig and Holman’s framework reveals that self-management includes three tasks [10]: medical management, role management, and emotional management. Medical management refers to the ability of patients to adopt behaviours beneficial to the disease in the process of disease management [23]. Medication adherence, exercises, and joint protection are recognized as vital aspects of medical management for RA patients. Role management means that patients with chronic diseases can properly undertake more activities, such as housework, employment work, having good social interactions, etc. [23]. Emotion management refers to the patient's ability to manage negative emotions (such as pain, sadness, depression, etc.) caused by the disease and reduce its negative life impact [23]. Meanwhile, according to this framework, there are five self-management skills that are central to patient self-management, including solving problems, making decisions, utilizing potential resources, formatting a patient-provider partnership, and taking action. Thus, this scale initially includes three tasks and nine aspects based on Lorig and Holman’s conceptual framework, a literature review and RA patients’ characteristics. Medical management includes six aspects: reasonable medication, self-monitoring, joint protection, exercise, diet, and healthy lifestyle. Role and emotional management includes rest and work, social interaction, and emotional management.

## Methods

### Design

The study was conducted in two phases: (1) scale development, and (2) psychometric evaluation: refining the scale and evaluating the psychometric properties.

### Phase 1 Scale development

#### Creating the item pool

The initial item pool of the scale was developed based on 2 sources: (1) a comprehensive literature review, and (2) findings from in-depth reviews.

In the literature review, we searched published articles in Web of Science, PubMed, Embase, EBSCO, CNKI, and WanFang Data. The keywords in the search were identified as “rheumatoid arthritis”, “self-care”, “self-care behaviour”, “self-management” and “self-management behaviour”. The inclusion criteria for the articles were: (1) relevant to RA patients’ self-management or focusing on self-management behaviours among patients with chronic disease; (2) quantitative and/or qualitative studies, and recommendations; and (3) published in English or Chinese. We retrieved a total of 2330 articles. After removing duplicates and screening by title and abstract, 356 articles (51 Chinese articles and 305 English articles) were included based on inclusion and exclusion criteria.

During the stage of the interview, face-to-face, semi-structured, in-depth individual interviews with 6 RA patients were performed. The primary questions in the interview asked were "How do you understand self-management behaviours?" and "What do you think is the content of self-management behaviours". The qualitative content analysis method was applied to analyse the review data.

#### Preliminarily evaluating the items

We used the NGT, Delphi expert consultation, and a pilot test to preliminarily evaluate and revise the items.

The NGT was used to evaluate and revise the initial item pool and dimensions by structuring face-to-face meetings with 9 experts to facilitate discussion and reach a consensus. The nine experts were invited from West China Hospital, Sichuan University. The inclusion criteria of the expert panel: (1) have practical experiences and/or theoretical knowledge of chronic diseases and rheumatic diseases self-management, (2) have intermediate or senior titles, (3) are willing to participate in our study. The NGT comprised 5 stages which included introducing and explaining the purpose and procedure of the meeting, carrying on a silent generation of ideas, presenting all ideas in a round-robin manner, clarification of any unclear ideas/items, and all participants voting on the importance of ideas [24]. We conducted 2 rounds of NGT to revise the instrument.

Two rounds of Delphi expert consultation were used to revise the items and dimensions, and improve this scale. We invited 21 experts experienced in rheumatology disease management for at least 10 years from 15 tertiary hospitals, and received responses from 20 experts. The inclusion criteria of the experts were the same as the NGT experts. The anonymous consultation questionnaires were sent to the experts by email. The positive coefficient, degree of authority, and coordination coefficient of experts were used to evaluate the results of the expert consultation. The positive coefficient of experts was assessed by the response rate [25]. An authority coefficient (Cr) of over 0.8 is considered a high expert authority coefficient [26]. For the coordination coefficient of experts, Kendall’s coefficients of concordance (Kendall’s W) of 0.5 or above is considered a high correlation, and *P* < 0.05 is considered statistically significant [27]. The coefficient of variation (CV) is an important basis for index deletion. Meeting the following conditions were retained: mean ≥ 4, SD < 1, and CV < 0.2. Meanwhile, experts’ input were also incorporated to modify the items and formulate a draft scale.

A pilot test was conducted after Delphi expert consultation. We recruited 20 RA patients to complete the draft scale through face-to-face interviews to check comprehensibility, readability, and response errors. Then, the researchers communicated with the participants and formulated an original scale based on the participants’ feedback and advice.

### Phase 2 Psychometric evaluation

In this stage, the original version of the Self-Management Behaviours Scale was used to evaluate the self-management behaviours of RA patients. We used item analysis, validity test and reliability to filter the items and evaluate the psychometric properties.

#### Item analysis

Item analysis was performed to determine whether each item in the original instrument should be retained or deleted. Low-quality items were removed from the scale.

#### Validity analysis

##### Content validity

Content validity was used to test content validity of the original scale. Seven additional experts agreed to participate in the consultation to evaluate the content validity of RA-SMBS. The inclusion criteria of experts were the same as the NGT experts. The experts reviewed the wording, comprehensiveness, and relevance of each item based on a 4-point scale [28]. The experts scored each item separately from 1 (not relevant) to 4 (very relevant and succinct). The Content Validity Index (CVI) based on expert ratings of relevance evaluated Content validity for the multi-item scale.

##### Construct validity

Exploratory Factor Analysis (EFA) and Confirmatory Factor Analysis (CFA) were used to estimate structural validity. The suitability of the data was evaluated with the Kaiser–Meyer–Olkin (KMO) coefficient and Bartlett's sphericity test. The EFA was conducted to extract factors by applying the principal components analysis with varimax rotation. The factor structure obtained from EFA was tested via CFA. Modification indices were evaluated to obtain a stronger model. The standardized factor loadings of < 0.50 should be deleted [29]. The model was rearranged by adding covariance between error terms. Goodness-of-fit was evaluated by using the chi-square minimum/degree of freedom (χ^2^/df), Root Mean Square Error of Approximation (RMSEA), Goodness of Fit Index (GFI), Comparative Fit Index (CFI), Incremental Fit Index (IFI), Tucker-Lewis Index (TLI), Parsimonious Normed Fit Index (PNFI) and Parsimonious Goodness-of-Fit Index (PGFI).

The convergent and discriminant validity of the scale were evaluated, and standardized factor loadings, average variance extracted (AVE), composite reliability (CR), and the square root of AVE of the scale were reported for the final model. Convergent validity can evaluate the level of correlation of multiple items of the same factor that are in agreement. Discriminant validity refers to the degree of difference between different latent variables and is valid if the correlation between latent variables is low.

#### Reliability analysis

The Cronbach's alpha coefficient and the split-half Spearman-Brown coefficient were used to assess the internal consistency and reliability of the scale and dimensions. The test–retest reliability was calculated to evaluate the stability of the scale. The test–retest reliability was evaluated by the intraclass correlation coefficients (ICC) and Pearson’s *r*. The ICC was assessed by a two-way mixed model with agreement.

### Participants

All patients were recruited from the Department of Rheumatology and Immunology, West China Hospital, Sichuan University between July 2020 to January 2022. RA patients were recruited to participate in in-depth individual interviews (*n* = 6), a pilot test (*n* = 20), psychometric evaluation (*n* = 561), and test–retest (*n* = 20). The patients’ inclusion criteria in the in-depth individual interviews, a pilot test, psychometric evaluation, and test–retest stage were: (1) diagnosed with RA based on the 2010 American College of Rheumatology criteria (ACR) / European League Against Rheumatism (EULAR) classification criteria; (2) diagnosed with RA for six months or more; (3) ≥ 18 years, and (4) able to speak and understand Chinese. We excluded the patients who had psychosis or serious primary diseases (e.g., heart, brain, liver, etc.), or who had other rheumatic diseases (e.g., systemic lupus erythematosus, Sjogren's syndrome, etc.). The Individual and Family Self-management Theory demonstrates that self-management is a process by which individuals use knowledge, belief, skill, and ability to achieve outcomes (e.g., self-management behavior) [30]. Self-management behaviour takes time to develop. Many previous studies selected RA patients with 6 months after diagnosis [21, 31, 32]. In the current study, we selected patients diagnosed with RA for six months or more. For the in-depth individual interviews, we used purposive sampling to recruit 6 RA patients, and all patients completed our interviews and were included in the data analysis. For the pilot test, a convenience sample of 20 patients was recruited, and all of them completed the survey and were included in the data analysis.

In the psychometric evaluation stage, regarding the required sample size in factor analysis, the sample size of exploratory factor analysis was calculated to be 8 participants per item of scale, which is equivalent to 280 participants in our study. As for confirmatory factor analysis, the appropriate sample size was 200 or more. The final sample size was 534, allowing a 10% dropout. Finally, a total of 580 questionnaires were distributed by convenient sampling and 561 RA patients completed the questionnaires, with an effective return rate of 96.72%. For the test–retest, a convenience sample of 20 questionnaires was collected.

### Data collection

Data were collected about the whole process. For the in-depth individual interviews, we recruited 6 RA patients and specified an appropriate place and date to conduct the interview. The interviews were recorded with a voice recorder. Each participant was interviewed once or twice and each interview lasted an average of 45–60 min. For the pilot study, we recorded patients’ comments about these items. In the psychometric test stage, the questionnaire took about 10–15 min to complete. The researchers explained the purpose of the study and the approach to completing the questionnaire. Participants completed the questionnaire independently or with the assistance of the researchers. Twenty participants who completed the questionnaire were invited to complete the questionnaire again after two weeks.

### Statistical analysis

The data were analysed using NVivo for windows version 12, the Statistical Package for Social Sciences (SPSS) for Mac version 25.0 and AMOS for Windows version 21.0. Descriptive statistics were used to describe the demographic characteristics of the participants, including numbers, frequency (%), mean, and SD.

For item analysis, if the corrected item-total correlation (CITC) was less than 0.30 and Cronbach’s alpha increased if the item was deleted, then these items were respectively removed from the scale [33].

For validity analysis, we conducted content validity and construct analysis. The I-CVI and S-CVI were calculated to determine the content validity of the scale items. The I-CVI value of more than 0.83, the S-CVI/UA value of more than 0.70 and the S-CVI/Ave value of more than 0.90 were considered as appropriate and acceptable [34]. The construct analysis included EFA, CFA, and the convergent and discriminant validity. The total sample was split into two sub-sample using the SPSS random-assignment function. Subsample 1 (281 samples) was used for EFA and subsample 2 (280 samples) was used for CFA. For EFA, the criterion for appropriate factor extraction was an eigenvalue of 1.00 or more, and the result was considered good when there was at least 60.00% of variance [35]. For CFA, model fit was considered acceptable if the χ^2^/df ratio was lower than 3, and with the GFI, CFI, IFI, and TLI above 0.90 meaning good fit and 0.80 meaning reasonable fit, the PNFI and PGFI above 0.50 meaning good fit, and the RMSEA below 0.05 meaning good fit and 0.05 to 0.08 meaning reasonable fit [36].

The convergent and discriminant validity of the scale was determined by calculating AVE, CR, and root AVE square. The formula is [37]:

- AVE = (sum of squared standardized loadings) / (sum of squared standardized loadings + sum of oberved variable measurement error).
- CR = (sum of standardized loadings)^2^ / ((sum of standardized loadings)^2^ + (sum of oberved variable measurement error)).

The convergent validity tests of AVE (> 0.5) [37] and CR (> 0.7) [38] confirmed that items of each factor were in agreement and accurately measured. The square root of AVE being greater than all the possible two-factor correlation coefficients (Ф) (AVE > Ф^2^) determined the discriminant validity between the two factors [37].

For reliability analysis, Cronbach’s alpha coefficients, and test–retest reliability coefficients were calculated by the total sample (561 samples) and the test–retest sample (20 samples). Cronbach’ s alpha coefficient above 0.80 indicates acceptable internal consistency reliability [39]. The ICC value of 0.70–1.00 was considered to have excellent stability, 0.60–0.70 as good stability, and 0.40–0.60 as reasonable stability, but below 0.40 as poor stability [40]. The Pearson’s r of more than 0.30 indicated good stability [41].

### Ethical considerations

The research was approved by West China Hospital Medical Ethics Committee in China (ID 2020997). Written informed content was obtained from participants in the in-depth individual interviews, pilot test, and psychometric test. The researchers informed the participants of the purpose and content of this study and obtained consent from all participants before the data collection. Participation was voluntary, and participants were guaranteed that they could withdraw from the study without stating any reasons. Data were ensured to be confidential and used for research purposes only.

## Results

### Scale development

A total of 186 items were screened by the literature review, of which 78 items were excluded as duplicates, and 64 items with similar meanings were merged. A pool of 44 items of RA patients’ self-management behaviour scale was initially formed. After adding in-depth interviews’ results, the initial item pool was composed of 46 items.

In the first round of NGT, we deleted 7 items, added 4 items, revised the remaining items, and retained 33 items. In the second round of NGT, we modified the scale into 6 dimensions, including medication management, symptom management, exercise, lifestyle management, resource utilization and social support, and emotional management (see Additional file 1).

During 2 rounds of Delphi consultations, 21 and 20 experts were invited respectively. The positive coefficients of experts were 95.2% and 100%. The Cr of the 2 rounds were 0.94 and 0.94. The Kendall’s W were 0.193 (*P* < 0.05) and 0.268 (*P* < 0.05) respectively. In the first round, 56 pieces of advices were proposed by these experts, including adding 11 items, removing 1 item, and modifying the wording of 11 items. Our research team discussed, revised, and formulated 39 items based on their expert advice. In the second round, 39 items were modified by deleting 2 items, merging 3 items to form an additional item, and adjusting the wording of 6 items based on the expert advice (see Additional file 1). Finally, we created a 35-item scale with a 5-point Likert scale for the pilot test.

In the pilot test, 20 RA patients completed the 35-item scale and shared their ideas about this scale. We modified the wording of three items (e.g., ‘Do not stay up late and overwork’ was revised to ‘Do not stay up late or overwork’, et al.) based on the participants’ feedback and improved the comprehensibility and accuracy of the items. Ultimately, the original scale comprised 35 items and 6 dimensions (see Additional file 2).

### Psychometric evaluation

#### General characteristics of participants

A total of 561 RA patients completed the questionnaires. The age of the participants ranged from 18 to 85 with a mean age of 47.88 (SD = 12.88). Most of the participants were female (520, 92.7%), living in cities (379, 67.6%) and never smoking (522, 93.1%). Around half of the participants had less than a high school education (295, 52.6%), and had joint deformation (283, 49.6%). The total sample (*n* = 561) was randomly classified into subsample 1 and subsample 2. The subsample 1 included 281 participants, and the subsample 2 included 280 participants. Participants’ characteristics are shown in Table 1.

**Table 1:** Demographic characteristics of the study participants

Variables	Total sample	Subsample 1	Subsample 2
	**(*N* = 561)**	**(*N* = 281)**	**(*N* = 280)**
	**n (%)**	**n (%)**	**n (%)**
**Gender**			
Female	520 (92.7)	268 (95.4)	252 (90.0)
Male	41 (7.3)	13 (4.6)	28 (10.0)
**Age (Years)**			
18 ~ 30	66 (11.8)	34 (12.1)	32 (11.4)
31 ~ 50	241 (43.0)	139 (49.5)	102 (36.4)
51 ~ 70	236 (42.0)	99 (35.2)	137 (48.9)
> 70	18 (3.2)	9 (3.2)	9 (3.2)
**Education**			
Bachelor’s degree or above	171 (30.5)	99 (50.9)	72 (25.7)
High school degree	95 (16.9)	48 (17.1)	47 (16.8)
Junior middle school degree	180 (32.1)	80 (28.5)	100 (35.7)
Primary school degrees or below	115 (20.5)	54 (19.2)	61 (21.8)
**Living**			
Cities	379 (67.6)	195 (69.4)	184 (65.7)
Towns and rural	182 (32.5)	86 (30.6)	96 (34.3)
**Smoking status**			
Never smoking	522 (93.1)	267 (95.0)	255 (91.1)
Yes, but having quit smoking	18 (3.2)	7 (2.5)	11 (3.9)
Yes, still smoking	21 (3.7)	7 (2.5)	14 (5.0)
**Joint deformation**			
Yes	283 (49.6)	152 (54.1)	149 (53.2)
No	278 (50.4)	129 (45.9)	131 (46.8)
	**Mean ± SD**	**Mean ± SD**	**Mean ± SD**
**Age (Years)**	47.88 ± 12.88	46.28 ± 12.47	49.49 ± 13.08

*SD* standard deviation

#### Item reduction

The total sample was used for the item analysis and the result identified four items (item 19, 23, 24, and 25) with the CITC of ≤ 0.30, removing these items resulted in an increase in the Cronbach’s alpha coefficient.

#### Content validity (CVI)

The content validity of the scale was assessed by the expert group. The I-CVI was more than 0.78, the S-CVI/Ave was 0.90, and S-CVI/UA had a value of 0.98. The results indicated the scale with good content validity.

#### Exploratory factor analysis

After the item analysis excluded the 19, 23, 24 and 25, the structural validity was performed using the 31 items. The subsample 1 was used for EFA. The results of the KMO coefficient (0.906) and Bartlett's test of sphericity (χ^2^ = 5675.967, df = 465, *P* < 0.001) were suitable for factor analysis. The EFA of the 31-item RA-SMBS was performed using principal component extraction and varimax rotation. The factor loading was less than 0.40, the difference of the cross-loadings was less than 0.1, and the number of common factors including items was less than 3 was eliminated to obtain a more robust factor structure. According to the excluded criteria of EFA and the research framework, seven items (items 11, 12, 22, 26, 27, 32, and 34) were deleted step by step through five rounds of factor analyses. Expert advice revealed that the scale demonstrated conceptual integrity after deleting the 7 items. For the remaining 24 items, although the difference between the cross-loadings of item 13 and item 21 was less than 0.1, their factor loadings were both above 0.40 and they played an important role in RA patients’ self-management behaviours. Item 13 and item 21 were retained based on the expert consultation. Finally, the RA-SMBS included 24 items with four factors. The KMO was 0.891 and Bartlett's test of sphericity was significant (χ^2^ = 4461.499, df = 276, *P* < 0.001). The factor loadings were 0.418 to 0.918. The four factors explained 61.89% of the total variance. The variances explained by each factor were 17.58%, 15.91%, 15.56%, and 12.84%, respectively (see Table 2).

**Table 2:** Exploratory factor analysis with data subsample 1

**Items**	**Mean ± SD**	**Factor 1 **	**Factor 2**	**Factor 3**	**Factor 4**	**Eigenvalues **	**Explained** ** Variation (%)**
**Item 1**	3.32 ± 1.02	**0.918 **	0.026	0.128	0.104	8.545	17.58
**Item 2**	3.27 ± 1.06	**0.915 **	0.070	0.099	0.101		
**Item 4**	3.51 ± 0.81	**0.887 **	0.101	0.148	0.047		
**Item 3**	3.18 ± 1.13	**0.776 **	0.203	0.077	0.172		
**Item 5**	2.67 ± 1.32	**0.675 **	0.238	0.245	0.094		
**Item 6**	2.85 ± 1.25	**0.550 **	0.352	0.103	0.160		
**Item 18**	2.65 ± 1.17	0.094	**0.857 **	0.169	0.145	2.777	15.91
**Item 17**	2.57 ± 1.25	0.108	**0.851 **	0.154	0.174		
**Item 15**	2.49 ± 1.27	0.153	**0.676 **	0.200	-0.024		
**Item 20**	2.98 ± 0.91	0.186	**0.670 **	0.070	0.132		
**Item 16**	1.62 ± 1.47	0.056	**0.564 **	0.364	0.162		
**Item 14**	2.51 ± 1.39	0.284	**0.535 **	0.268	0.281		
**Item 21**	2.67 ± 1.24	0.078	**0.451 **	0.357	0.177		
**Item 29**	1.92 ± 1.33	0.036	0.132	**0.734 **	0.188	1.885	15.56
**Item 28**	2.32 ± 1.34	0.148	0.119	**0.695 **	0.283		
**Item 33**	2.41 ± 1.25	0.066	0.196	**0.692 **	0.018		
**Item 30**	2.11±1.38	0.114	0.189	**0.692 **	0.213		
**Item 31**	1.63 ± 1.44	0.153	0.119	**0.653 **	0.127		
**Item 35**	3.07 ± 0.79	0.164	0.181	**0.588 **	0.007		
**Item 13**	2.91 ± 1.31	0.208	0.352	**0.418 **	0.163		
**Item 8**	2.89 ± 1.09	0.180	0.149	0.209	**0.899 **	1.647	12.84
**Item 7**	2.89 ± 1.10	0.186	0.158	0.201	**0.887 **		
**Item 9**	2.60 ± 1.31	0.131	0.203	0.123	**0.837 **		
**Item 10**	2.37 ± 1.30	0.060	0.209	0.393	**0.537 **		
**Total ** **scale**							61.89

**Note: **Factor 1, medication management; Factor 2, exercise and joint protection; Factor 3, resource utilization and emotional management; Factor 4, symptom management

The original six factors were compared with the final four factors, also with the consideration of the conceptual framework for self-management. The names of Factor 1 and Factor 4 were ‘medication management’ and ‘symptom management’, respectively. Factor 2 was named ‘exercise and joint protection, and Factor 3 was named ‘resource utilization and emotional management.

### Confirmatory factor analysis

**Fig. 1:**
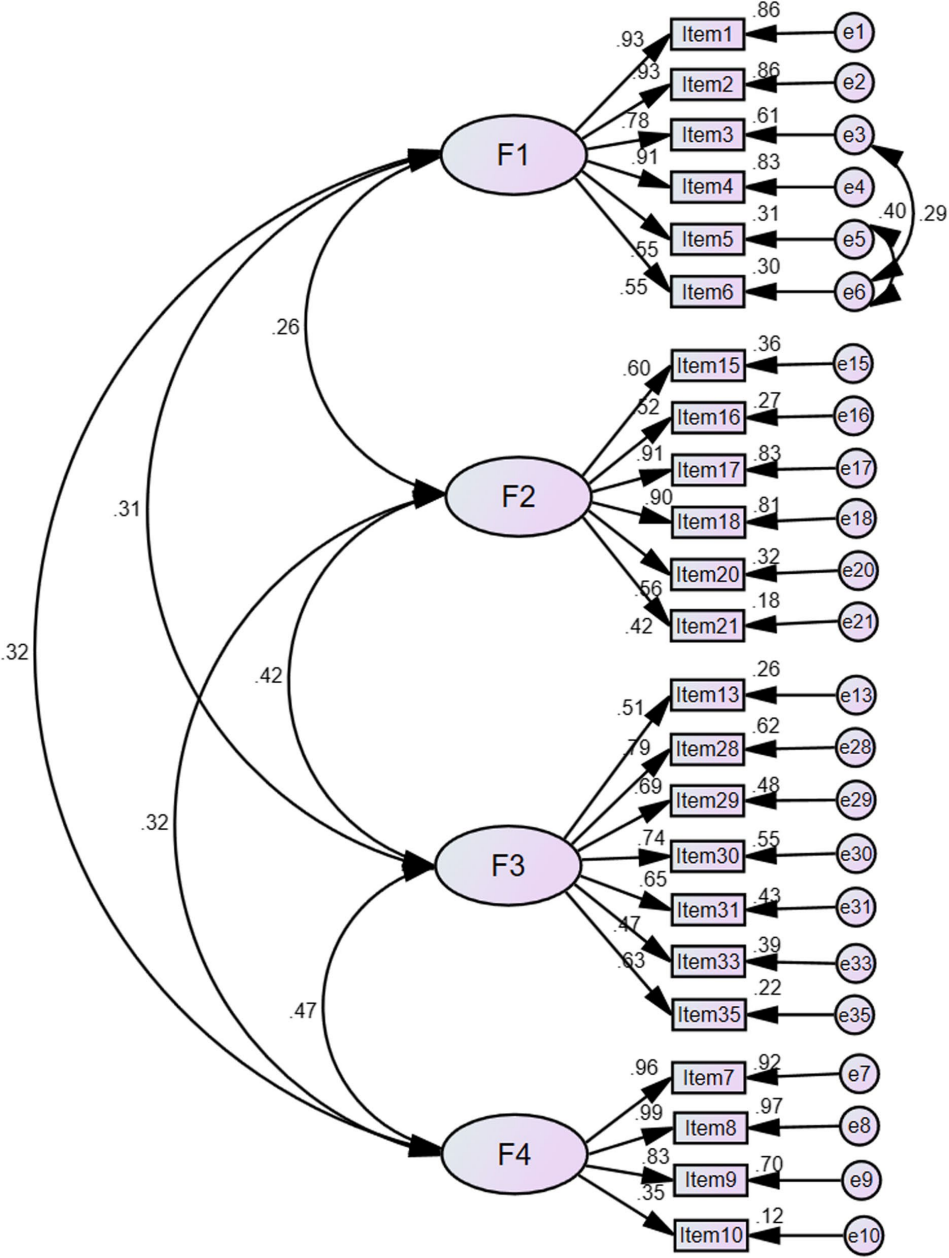
Confirmatory factor analysis of the four-factor model of the RA-SMBS. Note: F1: medication management; F2: exercise and joint protection; F3: resource utilization and emotional management; F4: symptom management

The 24-item scale with 4 dimensions which was found with EFA was tested with CFA. The subsample 2 was used for CFA. The standardized factor loadings of items 10, 14, 21 and 35 were 0.353, 0.439, 0.442 and 0.471, less than 0.5. We considered removing item 14 and remained items 10, 21, and 31, as the three items (items 10, 21, and 31) are closely related to self-management of patients with RA, and item 14 is not applicable for patients with mild symptoms. Finally, we re-examined the model fit of the 23-item scale after excluding item 14 (see Fig. 1). A high correlation was found between item 3 and item 6, item 5 and item 6. Thus, we added the error covariance to the model. Finally, the fit model of CFA was acceptable: χ^2^ = 588.788 (df = 222, *P* < 0.001), χ^2^/df = 2.652, RMSEA = 0.077 (90%CI = 0.069-0.085, *P* < 0.001), GFI = 0.845, IFI = 0.909, TLI = 0.895, CFI = 0.908, PGFI = 0.680, PNFI = 0.756 (see Table 3).

**Table 3:** Confirmatory factor analysis with data subsample 2

Fit index	χ^2^	df (*p*)	χ^2^/df	RMSEA (90% CI)	GFI	IFI	TLI	CFI	PGFI	PNFI
Model of RA-SMBS	588.788	222 (< 0.001)	2.652	0.077 (0.069–0.085)	0.845	0.909	0.895	0.908	0.680	0.756
Reference value										
Acceptable			< 5	< 0.08	> 0.90	> 0.90	> 0.90	> 0.90	> 0.50	> 0.50
Good			< 3	< 0.05	> 0.95	> 0.95	> 0.95	> 0.95	> 0.90	> 0.90

*X2* chi-square; df, degrees of freedom, *CI* confidence interval, *RMSEA* root mean square error analysis, *GFI* goodness of fit index, *IFI* incremental fit index, *TLI* Tucker-Lewis index, *CFI* comparative fit index, *PGFI* parsimonious goodness-of-fit index, *PNFI* parsimonious normed fit index

### Convergent and discriminant validity

The results of the convergent validity analysis showed that the standardized factor loading values of the 23-item scale ranged from 0.510 to 0.986 except for item 10 (0.353), item 21 (0.423), and item 35 (0.470). The CR values ranged from 0.826 to 0.972 and the AVE values from 0.422 to 0.678. The convergent validity was acceptable. Although the AVE of Factor 2 and Factor 3 are slightly less than 0.5, they achieved acceptable values of CR. The square roots of the AVE were greater than correlations between dimensions of the scale, and it indicated low related between the dimensions and other dimensions, and reasonable discriminant validity of the scale (see Table 4).

**Table 4:** Convergent and discriminant validity, Internal consistency reliability and test–retest reliability

	**Convergent and discriminant validity**			**Internal consistency reliability**		**test** **–** **retest reliability**	
**Total scale/ Dimensions**	**Sted**	**CR** **(> 0.7)**	**AVE** **(> 0.5)**	**Cronbach’s alpha**	**The split-half Spearman-Brown coefficient**	**ICC**	**Pearson’s r**
**Item 1 ← F1**	0.927	0.907	0.628	0.898	0.855	0.760**	0.767**
**Item 2 ← F1**	0.930						
**Item 4 ← F1**	0.782						
**Item 3 ← F1**	0.911						
**Item 5 ← F1**	0.554						
**Item 6 ← F1**	0.546						
**Item 15 ← F2**	0.600	0.826	0.462	0.822	0.780	0.430*	0.511*
**Item 16 ← F2**	0.520						
**Item 17 ← F2**	0.911						
**Item 18 ← F2**	0.901						
**Item 20 ← F2**	0.564						
**Item 21 ← F2**	0.423						
**Item 13 ← F3**	0.510	0.832	0.422	0.826	0.758	0.503*	0.676**
**Item 28 ← F3**	0.790						
**Item 29 ← F3**	0.690						
**Item 30 ← F3**	0.743						
**Item 31 ← F3**	0.653						
**Item 33 ← F3**	0.626						
**Item 35 ← F3**	0.470						
**Item 7 ← F4**	0.959	0.884	0.678	0.860	0.836	0.399*	0.574**
**Item 8 ← F4**	0.986						
**Item 9 ← F4**	0.835						
**Item 10 ← F4**	0.353						
**Total scale**				0.908	0.788	0.628**	0.780**

** *P*-value < 0.01

* *P*-value < 0.05

### Reliability analysis

The Cronbach’s alpha coefficient of the 23-item scale was 0.908. The Cronbach's alpha coefficients of the dimensions ranged from 0.822 to 0.898. The split-half Spearman-Brown coefficient of 0.788 further confirmed the internal consistency and reliability of the RA-SMBS (see Table 4).

In the results of test–retest reliability, ICC of the 23-item scale was 0.628 (*P* < 0.001), and ICC of the dimensions were 0.760 (*P* < 0.001), 0.430 (*P* = 0.026), 0.503 (*P* = 0.010), and 0.399 (*P* = 0.037). The Pearson’s *r* of the 23-item scale was 0.780. The dimensions of Pearson’s *r* were 0.767, 0.511, 0.676, and 0.574, respectively (see Table 4).

### Final scale

The RA-SMBS includes 23 items and 4 dimensions: medication management, symptom management, exercise and joint protection, and resource utilization and emotional management (see Additional file 3).

## Discussion

This study aimed to develop and evaluate the psychometric properties of a self-reported RA-SMBS. The results showed that this questionnaire had good validity, reliability, and internal consistency, indicating this scale can be used to evaluate RA patients’ self-management behaviours. In general, the psychometric properties of RA-SMBS are more reliable compared to other self-management scales for RA patients [17, 19–21]. It may be that this scale was developed based on Lorig and Holman’s conceptual framework, a literature review, in-depth reviews, the NGT, and Delphi expert consultation.

The distribution of demensions is roughly the same as the three tasks and of Lorig’s self-management. Medication management, symptom management, exercise and joint protection reflect the first task of medical management. RA patients are often present with joint pain, swelling, and morning stiffness [3], required long-term medication to achieve remission or near remission, and required self-monitoring of symptoms, as well as synovitis erosion of joints that could lead to joint damage and functional limitations [42]. Thus, the three dimensions are of major importance in patients with RA. Resource utilization and emotional management reflect tasks of role management, and emotional management. Based on the three tasks, the Lorig and Holman’s five self-management skills complement other content such as utilizing social resource, seeking help with doctors, family and friends, and furthering efforts to protect joints.

According to RA disease characteristics, the three aspects of medication management, symptom monitoring, and joint exercise are critical aspects to address in the treatment, they are also significant indicators of the ability of self-management behaviours. Meanwhile, access to medical and social support, building partneiships, and emotion regulation are also intergral to their self-management abilities. Long-term treatment increases the psychological and economic burden of patients [43, 44]. Studies show that the prevalence of depression among RA patients varies between 9.5–41.5% [45], which would become a stumbling block in the treatment process. Besides, emotional management is an essential component of support for patients with chronic diseases. Reasonable utilization of medical and social resources could enhance patients’ confidence in treatment adherence [46], which is also a manifestation of patients’ improved self-management ability.

The dimension of medication management with six items addresses patients’ knowledge of medication use and their medication adherence. The SCBS among patients with RA was developed by Nadrian, et al. in 2019. It included the dimension of medication (three items), but while two items are inverse to each other, the three items do not reflect well on the ability of patients to manage medications. The items of joint protection were not grouped into the same dimension but were assigned to ‘nutrition/joints protection’ and ‘stress management/others’, respectively [21]. There is a lack of reasonable explanations for such a distribution of dimensions, and that is considered a weakness of their study. Also, the researchers relied solely on a literature review and expert consultation when developing the items of the SCBS without consideration of the experience and perspectives of patients. Our study, however, had a more comprehensive process of item development with the integration of Lorig and Holman’s conceptual framework, the NGT, and in-depth interviews. Strengths of the present study specifically included using face-to-face expert consultations and gathering participants' feedback, which further contributed to the successful development of items and increased reliability of the results. Additionally, the items of the RA-SMBS were developed according to the characteristics of patients, the scale demonstrates good psychological properties which also make it very practical.

## Limitations

This study has several limitations. Firstly, criterion validity was not assessed in the study due to the lack of a golden criterion to evaluate RA patients’ self-management behaviours. Secondly, all of the participants were recruited from a tertiary hospital in Chengdu. The single-center study design could limit the generalizability of the findings. Future research is needed to examine the psychometric properties of RA-SMBS in other populations or settings in which the construct is applied. Thirdly, the test–retest reliability ICC of a dimension was not adequate. Future studies could seek to involve more participants with different phases of RA development. Fourth, we did not evaluate the self-management behaviours of RA patients using the developed scale due to limited time. We will validate the RA-SMBS in future studies. Finally, future research endeavours should also strive to include a large representative sample and further test the model fit and the convergent validity.

## Conclusion

This study developed a 23-item RA-SMBS with 4 dimensions. Our results revealed that the RA-SMBS is a reliable and valid scale to evaluate RA patients’ self-management behaviours. The RA-SMBS has a score distribution ranging from 0 to 92, with higher scores indicating higher self-management behaviours. The RA-SMBS can be applied as an effective instrument to evaluate RA patients’ self-management behaviours in clinical settings and the development of self-management educational programs for RA patients. The Chinese version of RA-SMBS is reliable and validated. However, it would require further testing in different populations. A translated version in any other language would also require appropriate validation.

## Supplementary Information

**Additional file 1.** 

**Additional file 2.** 

**Additional file 3.**

## Data Availability

The datasets used and/or analysed during the current study are available from the corresponding author on reasonable request.

## Abbreviations

RA-SMBS: Rheumatoid Arthritis Self-Management Behaviours Scale
RA: Rheumatoid Arthritis
SCBS: Self-Care Behaviours Scale
CDSMS: Chronic Disease Self-Management Study Measures
PAM: The Patient Activation Measure
SMAS-S: The Short Self-Management Ability Scale
CNKI: China Network Knowledge Infrastructure
ACR: American College of Rheumatology
EULAR: European League Against Rheumatism
NGT: The Nominal Group Technique
Cr: The authority coefficient
Kendall’s W: Kendall’s coefficients of concordance
CV: The coefficient of variation
SD: Standard Deviation
CITC: The Corrected Item-Total Correlation
I-CVI: The Item-level Content Validity Index
S-CVI: The Scale-level Content Validity Index
EFA: Exploratory Factor Analysis
CFA: Confirmatory Factor Analysis
KMO: The Kaiser–Meyer–Olkin
χ^2^/df: Chi-square minimum/Degree of Freedom
RMSEA: Root Mean Square Error of Approximation
GFI: Goodness of Fit Index
CFI: Comparative Fit Index
IFI: Incremental Fit Index
TLI: Tucker-Lewis Index
PNFI: Parsimonious Normed Fit Index
PGFI: Parsimonious Goodness-of-Fit Index
AVE: Average Variance Extracted
CR: Composite Reliability
Ф: The possible two-factor correlation coefficients
ICC: The Intraclass Correlation Coefficients
SPSS: Statistical Package for Social Sciences
