# Supplementary material for: Development and psychometric evaluation of a self-management behaviours scale in rheumatoid arthritis patients (RA-SMBS)
Source: BMC Nurs. 2023 Feb 14;22:40. doi: 10.1186/s12912-023-01173-4 (PMC9926751; doi:10.1186/s12912-023-01173-4)
Supplement: Supplementary file 1 — Additional file 1. [file 12912_2023_1173_MOESM1_ESM.docx]

**Additional files**

**Additional file 1:**

**The comments of the NGT and Delphi expert consultation**

**The details information** on the number of items and dimensions revised or deleted from **the NGT and Delphi expert consultation:**

| **The Nominal Group Technique (NGT)** | | |
| --- | --- | --- |
| **The**  **first round** | **Deleted**  **7 items** | 1. Item 7: Take supplements containing fish oil or omega-3 without consulting your physician  2. Item 15: Use joint protection, bracing, or splinting  3. Item 17: Use of complementary therapies (e.g., massage, physiotherapy, etc.)  4. Item 25: Use a heated pool, tub, or shower  5. Item 32: Use some facilities (like handles, armchair and so on) in toilet, bed room and bathroom to ease the processes of sitting down, standing up and walking  6. Item 34: Drinking  7. Item 40: Talk with persons who are sympathetic |
|  | **Revised items** | 1. The dimensions of "reasonable medication" and "self-monitoring" were merged into "medication management".  2. The two dimensions of "diet" and "healthy lifestyle" were modified as "lifestyle management".  3. Item 33 "smoking" was modified as "no smoking".  4. Item 1 "Know the effects and side effects of your drugs", item 5 "Changed the time of taking your drugs without informing your physician" and item 6 "Taking medication with meals to relieve stomach problems" were merged into a new item 1 "Know information about the drugs you are taking (e.g., effects and side effects, usage and dosage, etc.)".  5. Item 2 "Take medications as prescribed", item 3 "Forgetting to take medications", and item 4 "Reducing or stopping medications by yourself" were merged into a new item (item 2) "Take medications at the right and long-time method as prescribed (including time, dosage and regimens)".  6. Item 8 "Timely visit to doctors for follow-up visits", item 9 "Regular follow-ups" and item 10 "Regular self-assessment of physical condition" were merged into a new item (item 5) "Have regular medical follow-ups".  7. Item 35 "I can work in a healthy manner" and item 37 "Ability to recover energy after rest" were revised as a new item (item27) "Ability to balance work and rest and to recover energy after rest".  8. Item 45 "I will get better" and item 46 "Confidence for a better future" were merged into a new item (item 35) "Keep a positive attitude toward disease". |
|  | **Added**  **4 items** | 1. A new item (item 3) "Consult your doctor before taking other medications (e.g., herbs, supplements, etc.) on your own".  2. A new item (item 4) "Visit your physician timely and adjust your medication if you have adverse events or symptoms of the medication".  3. A new item (item 30) "Communicate with others (e.g., patients, etc.) to share feelings and discuss disease information".  4. A new item (item 32) "Actively participate in social activities and maintain good social relationships (e.g., public welfare activities, etc.)". |
|  | **Results** | The result retained 33 items in the first round of the NGT. |
| **The second round** | **Revised items** | No |
|  | **Revised dimensions** | 1. Added a new dimension "symptom management".  2. The dimension of "joint protection" and "exercise" were merged into "exercise".  3. All items of "rest and work" were merged into the dimension of "lifestyle management".  4. The dimension of "social interaction" was revised as "resource utilization and social support", and added relevant items. |
|  | **Results** | **The result formed 6 dimensions and 33 items by the two round of the NGT.** |
| **Delphi expert consultation** | | |
| **The**  **first round** | **Deleted**  **1 items** | **1. Lifestyle management:** deleted the item "Take measures to avoid wet environments". |
|  | **Added**  **11 items** | **Medication management:**  1. A new item "Contact your healthcare providers if you have any questions about the medication you are taking, such as its proper use, schedules, and doses".  2. A new item "When adjusting medication (e.g., adding or reducing dosage, stopping medication) under the guidance of your healthcare providers".  **Symptom management:**  3. A new item "Seek prompt medical attention if new-onset discomfort other than joint pain and swelling occurs".  **Exercise:**  4. A new item "Stay in bed when severe joint pain occurs, take precautions to limit joint compression, avoid pressure injury".  5. A new item "Have exercise therapy (e.g., joint exercises) tailored to individuals needs and under the guidance of health professionals".  6. A new item "Gradually increase the intensity of exercise, to avoid over-exercising and excess your tolerance".  **Lifestyle management:**  7. A new item "Have good quality protein at three meals".  8. A new item "Have fresh vegetables and fruit per meals".  9. A new item "Carbohydrate ration per meals".  10. A new item "Have dietary supplements containing calcium".  **Resource utilization and social support:**  11. A new item "Obtain timely social support when coping with the disease (e.g., health insurance, social assistance policy, public welfare support, etc.)". |
|  | **Revised items** | **Medication management:**  1. Item 2 "Take medications at the right and long-time method as prescribed (including time, dosage and regimens)" was revised as "Adhere to the prescribed medication regimens".  2. Item 4 "Visit your physician timely and adjust your medication if you have adverse events or symptoms of the medication" was revised as "Manage adverse events of the medication under the guidance of rheumatologists or nurses".  **Symptom management:**  3. Item "Observe signs of swelling and tenderness" was divided into two items "Observe signs of swelling" and "Observe signs of tenderness".  4. Item "Observe the duration and severity of morning stiffness" was revised as "Observe the duration of morning stiffness".  Item "Apply measures to relieve pain, morning stiffness, and other discomforts (e.g., soaking in hot water or apply heat to the affected joints)" was revised as "Apply measures to relieve pain, morning stiffness, and other discomforts (e.g., take moderate exercise or apply heat to the affected joints)".  5. Item "Consult physician when having significant changes in your medical condition (e.g., increased pain)" was revised as "Consult healthcare professionals promptly when having significant changes in your medical condition (e.g., increased pain); continue health monitoring if having minor changes".  6. Item "Have regular medical follow-up visits" was revised as "Have regular medical follow-up visits and keep your own medical records" and it was placed in dimension "symptom management".  **Exercise:**  7. Item "Choose a suitable exercise to train your joints or muscles (e.g., jogging, walking, cycling, Tai Chi, etc.)" was revised as "Take part in daily exercise tailored to individuals needs and under the guidance of health professionals (e.g., jogging, walking, cycling, Tai Chi, etc.)".  8. Item "Exercise 3-5 times a week", "Exercise at least 30 minutes per time" and "Tailor exercise to individual physical condition, or stop exercising" were merged into "Tailor exercise to individual physical condition (exercise approach, exercise time, amount of exercise, etc.), stop exercising when necessary (such as having dizziness, nausea, chest pain, or other discomforts)".  **Lifestyle management:**  9. Item "Balance activity and rest to maintain adequate energy" and item "Take measures to relieve fatigue when you feel it (e.g., rest for longer or promote sleep)" were merged into "Have regular daily routines, avoid staying up late and overwork, and ensure adequate sleep" and it was placed in "lifestyle management".  10. Item "Take measures to protect your joints (e.g., lift heavy objects with your arms instead of your fingers, drag objects without lifting them, etc." and item "Keeping warm in life (e.g., wear gloves when exposed to cold to keep your joints warm, etc.)" were merged into "Take measures to protect your joints (e.g., lift heavy objects with your arms instead of your fingers, wear gloves when exposed to cold to keep your joints warm, etc.)".  **Resource utilization and social support:**  11. Item "Learning proactively about disease-related health care through the internet, books, etc." was revised as "Engage with an active learning approach to obtain accurate disease-related knowledge". |
|  | **Results** | **The result retained 39 items through the first round of Delphi expert consultation.** |
| **The second round** | **Deleted**  **2 items** | 1. Deleted the item "When adjusting medication (e.g., adding or reducing dosage, stopping medication) under the guidance of your healthcare providers".  2. Deleted the item "Carbohydrate ration per meals". |
|  | **Revised items** | **Medication management:**  1. Item "Adhere to the prescribed medication regimens" was revised as "Adhere to the prescribed medication regimens (take the full course of the medication and stop the medication only with the doctor’s approval)".  2. Item "Manage adverse events of the medication under the guidance of rheumatologists or nurses" was revised as "Manage adverse events of the medication under the guidance of rheumatologists or nurses (such as timely consultation with doctors or nurses and making medical adjustments when developing symptoms such as nausea, abdominal pain, and skin rashes)".  **Symptom management:**  3. Item "Observe signs of swelling" was revised as "Observe signs of swelling (such as the location of swollen joints and number of joints affected etc.)".  4. Item "Observe signs of tenderness" was revised as "Observe signs of tenderness (such as the location of tender joints, duration, the intensity of tenderness, etc.)".  **Exercise:**  5. Item "Stay in bed when severe joint pain occurs, take precautions to limit joint compression, avoid pressure injury" was revised as "Stay in bed when severe joint pain occurs, take precautions to limit joint compression, avoid pressure injury and venous thrombosis of the lower limbs".  6. Item "Gradually increase the intensity of exercise, to avoid over-exercising and excess your tolerance" was revised as "Gradually increase the intensity of exercise and avoid over-exercising. Indicators of appropriate exercise intensity include the absence of increased joint discomfort (e.g., pain, swelling, etc.) and emotional instability".  **Lifestyle management:**  7. Item "Have good quality protein at three meals", item "Have fresh vegetables and fruit per meals" and item "Have dietary supplements containing calcium" were merged into a new item "Increase intake of fresh fruits, vegetables, nuts, and high-quality protein (e.g., fish, lean meat, legumes/beans, etc.), and have dietary supplements containing calcium". |
|  | **Results** | **Finally, the scale comprised of 35 items and 6 dimensions.** |
