# Supplementary material for: Development and psychometric evaluation of a self-management behaviours scale in rheumatoid arthritis patients (RA-SMBS)
Source: BMC Nurs. 2023 Feb 14;22:40. doi: 10.1186/s12912-023-01173-4 (PMC9926751; doi:10.1186/s12912-023-01173-4)
Supplement: Supplementary file 2 — Additional file 2. [file 12912_2023_1173_MOESM2_ESM.docx]

**Additional file 2:**

**Rheumatoid Arthritis Self-Management Behaviors Scale (RA-SMBS) (35 items)**

**Instructions for filling in:** The following questions describe the patients’ self-management behaviors. Please read each question carefully, and choose the one that best suits your situation from the 5 alternative answers based on your actual situation **within the last month**. And tick "√" in the corresponding form. There is no right or wrong answer, if you are not sure about the answer to a question, just choose the option that best fits your situation. There is one and only one answer for each question, please do not select multiple or omit.

| **Dimensions** | **No.** | **Items** | **Never** | **Occasionally** | **Sometimes** | **Often** | **Always** |
| --- | --- | --- | --- | --- | --- | --- | --- |
| **Medication management** | **1** | Take medications each time as the prescribed dose |  |  |  |  |  |
|  | **2** | Adhere to the medication dosing schedule as prescribed |  |  |  |  |  |
|  | **3** | Take medications at the right method as prescribed (e.g., before, with, or after a meal) |  |  |  |  |  |
|  | **4** | Adhere to the prescribed medication regimens (take the full course of the medication and stop the medication only with the doctor’s approval) |  |  |  |  |  |
|  | **5** | Contact your healthcare providers if you have any questions about the medication you are taking, such as its proper use, schedules, and doses. |  |  |  |  |  |
|  | **6** | Manage adverse events of the medication under the guidance of rheumatologists or nurses (such as timely consultation with doctors or nurses and making medical adjustments when developing symptoms such as nausea, abdominal pain, and skin rashes) |  |  |  |  |  |
| **Symptom management** | **7** | Observe signs of swelling (such as the location of swollen joints and number of joints affected etc.) |  |  |  |  |  |
|  | **8** | Observe signs of tenderness (such as the location of tender joints, duration, the intensity of tenderness, etc.) |  |  |  |  |  |
|  | **9** | Observe the duration of morning stiffness |  |  |  |  |  |
|  | **10** | Apply measures to relieve pain, morning stiffness, and other discomforts (e.g., take moderate exercise or apply heat to the affected joints) |  |  |  |  |  |
|  | **11** | Seek prompt medical attention if new-onset discomfort other than joint pain and swelling occurs |  |  |  |  |  |
|  | **12** | Consult healthcare professionals promptly when having significant changes in your medical condition (e.g., increased pain); continue health monitoring if having minor changes |  |  |  |  |  |
|  | **13** | Have regular medical follow-up visits and keep your own medical records |  |  |  |  |  |
| **Exercise** | **14** | Stay in bed when severe joint pain occurs, take precautions to limit joint compression, avoid pressure injury and venous thrombosis of the lower limbs |  |  |  |  |  |
|  | **15** | Take part in daily exercise tailored to individuals needs and under the guidance of health professionals (e.g., jogging, walking, cycling, Tai Chi, etc.) |  |  |  |  |  |
|  | **16** | Have exercise therapy (e.g., joint exercises) tailored to individuals needs and under the guidance of health professionals |  |  |  |  |  |
|  | **17** | Tailor exercise to individual physical condition (exercise approach, exercise time, amount of exercise, etc.), stop exercising when necessary (such as having dizziness, nausea, chest pain, or other discomforts) |  |  |  |  |  |
|  | **18** | Gradually increase the intensity of exercise and avoid over-exercising. Indicators of appropriate exercise intensity include the absence of increased joint discomfort (e.g., pain, swelling, etc.) and emotional instability |  |  |  |  |  |
| **Lifestyle management** | **19** | Have regular daily routines, avoid staying up late and overwork, and ensure adequate sleep |  |  |  |  |  |
|  | **20** | Tailor the schedules, amount, and content of paid work and unpaid work (including housework) to the individual physical condition |  |  |  |  |  |
|  | **21** | Take measures to protect your joints (e.g., lift heavy objects with your arms instead of your fingers, wear gloves when exposed to cold to keep your joints warm, etc.) |  |  |  |  |  |
|  | **22** | Increase intake of fresh fruits, vegetables, nuts, and high-quality protein (e.g., fish, lean meat, legumes/beans, etc.), and have dietary supplements containing calcium |  |  |  |  |  |
|  | **23** | Keep your weight in the normal range (BMI: 18.5-23.9, BMI = weight (in kg)/ height^2 (in m^2). |  |  |  |  |  |
|  | **24** | Keep an eye on weather changes and dress appropriately to avoid catching a cold |  |  |  |  |  |
|  | **25** | Remain a nonsmoker |  |  |  |  |  |
| **Resource utilization and social support** | **26** | Engage with an active learning approach to obtain accurate disease-related knowledge |  |  |  |  |  |
|  | **27** | Take an active part in health education training programs provided by rheumatologists (e.g., doctors, nurses) |  |  |  |  |  |
|  | **28** | Prepare a list of questions before the outpatient follow-up visit with the doctor |  |  |  |  |  |
|  | **29** | Communicate with others (e.g., healthcare professionals, patients, etc.) to share feelings and discuss disease information |  |  |  |  |  |
|  | **30** | Seek help and support from family or friends to work through difficulties in managing the disease |  |  |  |  |  |
|  | **31** | Obtain timely social support when coping with the disease (e.g., health insurance, social assistance policy, public welfare support, etc.) |  |  |  |  |  |
|  | **32** | Actively participate in social activities and maintain good social relationships (e.g., public welfare activities, etc.) |  |  |  |  |  |
| **Emotional management** | **33** | Take measures to manage emotions when you are feeling down (e.g., listening to music, etc.) |  |  |  |  |  |
|  | **34** | Seek professional help and psychological counseling when needed to reduce psychological pressure |  |  |  |  |  |
|  | **35** | Keep a positive attitude toward disease |  |  |  |  |  |
